# Rational modular design of metabolic network for efficient production of plant polyphenol pinosylvin

**DOI:** 10.1038/s41598-017-01700-9

**Published:** 2017-05-03

**Authors:** Junjun Wu, Xia Zhang, Yingjie Zhu, Qinyu Tan, Jiacheng He, Mingsheng Dong

**Affiliations:** 1 College of Food Science and Technology, Nanjing Agricultural University, Nanjing, Jiangsu 210095 China; 2 Institute of Agro-Product Processing, Jiangsu Academy of Agricultural Sciences, Nanjing, Jiangsu 210095 China

## Abstract

Efficient biosynthesis of the plant polyphenol pinosylvin, which has numerous applications in nutraceuticals and pharmaceuticals, is necessary to make biological production economically viable. To this end, an efficient *Escherichia coli* platform for pinosylvin production was developed via a rational modular design approach. Initially, different candidate pathway enzymes were screened to construct de novo pinosylvin pathway directly from D-glucose. A comparative analysis of pathway intermediate pools identified that this initial construct led to the intermediate cinnamic acid accumulation. The pinosylvin synthetic pathway was then divided into two new modules separated at cinnamic acid. Combinatorial optimization of transcriptional and translational levels of these two modules resulted in a 16-fold increase in pinosylvin titer. To further improve the concentration of the limiting precursor malonyl-CoA, the malonyl-CoA synthesis module based on clustered regularly interspaced short palindromic repeats interference was assembled and optimized with other two modules. The final pinosylvin titer was improved to 281 mg/L, which was the highest pinosylvin titer even directly from D-glucose without any additional precursor supplementation. The rational modular design approach described here could bolster our capabilities in synthetic biology for value-added chemical production.

## Introduction

The polyphenol pinosylvin (*trans*-3,5-dihydroxystilbene) is a plant secondary metabolite fulfilling several functions including protection from attack by microbes or insects^1^. Pinosylvin has emerged as a promising nutraceutical or pharmaceutical because of its antioxidative, anti-inflammatory, anticancer, and chemopreventive activities^2^. Pinosylvin is primarily existed in the heartwood of genus *Pinus*. However, the concentration of this compound in plants only ranges from 1 to 40 mg/g^3^, rendering access to this medicinally important product through plant extraction difficult. Additionally, isolation of single compound from plants is limited by seasonal and regional variations and often difficult due to the complexity of secondary metabolites from plant extracts^4^. Alternatively, microbial production of pinosylvin may accelerate its large-scale production and is more environmentally friendly^5^.

**Figure 1:**
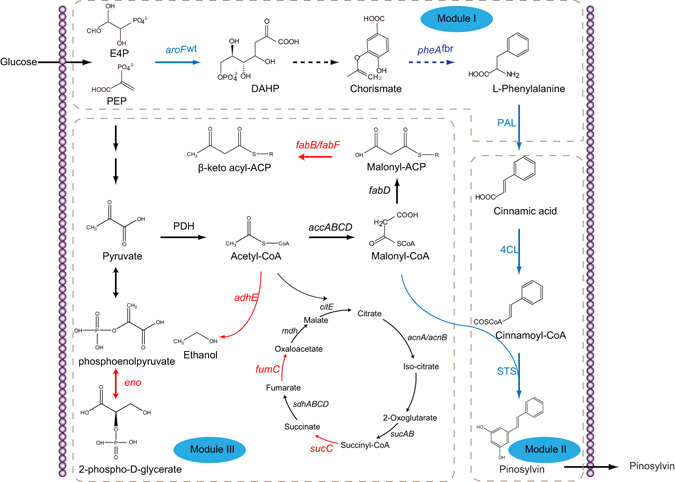
Schematics of the three modules. Module one consisted of *aroF* ^wt^, *pheA* ^fbr^ and TcPAL; Module two consisted of 4CL and STS; Module three consisted of CRISPRi to redirect endogenous central metabolism. The repressing genes are shown in red. The overexpressing genes are shown in blue. E4P meant erythrose-4-phosphate; PEP meant phosphoenolpyruvate; *aroF* meant the gene encoding 3-deoxy-D-arabinoheptulosonate-7-phosphate (DAHP) synthase; *pheA* ^fbr^ meant the gene encoding feedback-inhibition-resistant (fbr) chorismate mutase/prephenate dehydratase (CM/PDT); PAL meant phenylalanine ammonia lyase; 4CL meant 4-coumarate:CoA ligase; STS meant stilbene synthase.

The conversion of the aromatic amino acid L-phenylalanine to pinosylvin requires three steps (Fig. 1). Firstly, phenylalanine ammonia lyase (PAL) deaminates L-phenylalanine to cinnamic acid. 4-Coumarate:CoA ligase (4CL) subsequently converts cinnamic acid into its corresponding coenzyme A ester cinnamoyl-CoA. The resulting cinnamoyl-CoA is then condensed with three units of malonyl-CoA via stilbene synthase (STS) to form the stilbene pinosylvin^4^.

To date, several studies have made significant achievements for microbial production of stilbene^4, 5^, particularly for synthesizing resveratrol^6, 7^. For example, relevant study reported that supplementation of 15 mM *p*-coumaric acid led to a high product titer of 2.3 g/L resveratrol in *Escherichia coli* ^6^. However, these strategies rely heavily on supplementation of phenylpropanoids as stilbene precursors, which is costly and present in few industrial processes.

In contrast, de novo microbial pinosylvin production makes biological production economically viable and would accelerate the application of pinosylvin as both nutraceutical and pharmaceutical. Previous studies have demonstrated the feasibility of microbial production of pinosylvin from D-glucose or glycerol. In one relevant study, researchers constructed different configurations of three-step pinosylvin biosynthetic pathway and up to 3 mg/L titer was achieved. Further addition of fatty acid synthesis inhibitor cerulenin increased product titer to 70 mg/L^4^. As cerulenin is expensive and cost prohibitive for large-scale fermentation process, another study examined using clustered regularly interspaced short palindromic repeats interference (CRISPRi) system to repress *fadD* gene, and product titer of 47.5 mg/L was obtained from glycerol^5^. Additionally, *Corynebacterium glutamicum* also has been engineered and 121 mg/L of pinosylvin and 158 mg/L of resveratrol was achieved in the presence of 25 μM cerulenin^8^.

Despite exciting achievements under these methods, there is a pressing need to develop more economically viable process with high productivity and yield. However, the most difficult part exists in finding and applying effective resolutions to overcome metabolic flux imbalances when implementing a heterologous pathway using non-natural substrates^9, 10^. De novo pinosylvin synthesis from D-glucose involves manipulating multi-gene pathways that are subjected to tight cellular regulation. Previous studies always engineered part of the overall pathway such as L-phenylalanine to pinosylvin or even from cinnamic acid^4, 5^, ignoring the balance of the overall pathway. It was hypothesized that one pathway bottleneck might be eliminated while another bottleneck might be brought in somewhere else along the pathway when examining only part of the pinosylvin pathway^11^. Furthermore, endogenous central metabolism still strongly competes and predominates for energy and carbon sources during the synthesis of malonyl-CoA synthesis, leaving only a few amounts for producing recombinant products^12, 13^.

More recently, we introduced a modular metabolic engineering strategy to balance resveratrol synthetic pathway and achieved 35 mg/L resveratrol from 3 mM L-tyrosine^14^. Despite the potential of modular metabolic engineering to significantly bolster the capabilities in synthetic biology^15^, this area still lacks a standard principle for module grouping and further optimization. Here, a rational modular design approach was developed for grouping and optimizing modules. Compared to our previous studies^14, 16, 17^, this rational modular design approach demonstrated that choosing separating node at cinnamic acid rather than previous cinnamoyl-CoA lead to a dramatic increase in final production titer. Final pinosylvin titers were improved to 281 mg/L, which represented the highest titer reported to date. This rational modular design approach provides a framework for module grouping and optimization and would expedite developing robust and efficient microbial cell factories for value-added chemical production.

## Results

### Design of de novo pinosylvin synthetic pathway

For de novo microbial production of pinosylvin, strains displaying enhancing ability for the synthesis of L-phenylalanine are required. In *E. coli*, two rate-limiting steps exist toward the synthesis of L-phenylalanine. The first one is the condensing erythrose 4-phosphate (E4P) and phosphoenolpyruvate (PEP) via 3-deoxy-D-arabinoheptulosonate 7-phosphate synthase (DAHP) synthase isozymes encoded by *aroG*, *aroF* and *aroH*. The second one is the conversion of chorismate (CHO) to phenylpyruvate (PPY) via chorismate mutase/prephenate dehydratase (CM/PDT). Previously, a β-2-thienylalanine-resistant *E. coli* K12 mutant exhibiting high titers of L-phenylalanine was obtained^18^. This strain carried a wild-type DAHP synthase (DAHPS: *aroF* ^*wt*^) and a mutant CM/PDT (CM/PDT: *pheA* ^*fbr*^). As such, *aroF* ^*wt*^ and *pheA* ^*fbr*^ were overexpressed to enhance L-phenylalanine synthesis^19^.

As the first step of phenylpropanoid pathway, two candidate PAL enzymes were chosen. One was selected from the red yeast *Rhodotorula glutinis* (RgPAL), which was successfully used in our previous studies^16, 19^. The other one was chosen from *Trichosporon cutaneum* (TcPAL), which was a novel phenylalanine/tyrosine ammonia-lyases exhibiting high activities toward both L-phenylalanine and L-tyrosine^20^. 4CL from *Petroselinum crispum* and STS from *Vitis vinifera* served as the second and third enzyme because these two enzymes achieved the highest production of stilbene resveratrol in *E. coli* demonstrated by previous study^6^.

### Analysis of pathway intermediate pools

Based on our previous study, *aroF* ^wt^ and *pheA* ^fbr^ under T7 promoter in pCOLADuet-1 (pCOLA-aroF^wt^-pheA^fbr^), RgPAL or TcPAL and 4CL under Trc promoter in pCDFDuet-1 (pCDFD-Trc-RgPAL-Trc-4CL or pCDFD-Trc-TcPAL-Trc-4CL), STS under T7 promoter in pETDuet-1 (pETD-STS) were overexpressed in *E. coli* BL21(DE3) strain to construct the initial fermentation platform, as this combination always led to the highest flavonoid^16, 21^ or stilbene titer^14^. We found that the combination containing TcPAL produced higher pinosylvin (10.5 mg/L) than RgPAL (3.7 mg/L). To confirm the TcPAL activity, the RgPAL and TcPAL were cloned into pCDFDuet-1 individually and it was found that *E. coli* with TcPAL produced higher cinnamic acid (466 mg/L) than RgPAL (249 mg/L) (Table 1). Hence, TcPAL was used for the following research.

**Table 1:** Analysis of intracellular pools of pathway intermediates.

Strain	Concentrations after 48 h (mg/L)			
	L-Phenylalanine	Cinnamic acid	*p*-Coumaric acid	Pinosylvin
pCDFD-T7-RgPAL^a^	99 ± 3	249 ± 4	27 ± 0.4	0
pCDFD-T7-TcPAL^b^	125 ± 3	465 ± 6	40 ± 0.3	0
pCDFD-Trc-RgPAL-Trc-4CL^c^	90 ± 2	200 ± 3	26 ± 0.3	3.7 ± 0.03
pCDFD-Trc-TcPAL-Trc-4CL^d^	116 ± 3	404 ± 6	36 ± 0.2	10.5 ± 0.11

^a^RgPAL was directly cloned into *Nco*I/*Avr*II sites of pCDFDuet-1. Engineered strains contained pCOLA-T7-aroF^wt^-T7-pheA^fbr^ and pCDFD-T7-RgPAL.

^b^TcPAL was directly cloned into *Nco*I/*Avr*II sites of pCDFDuet-1. Engineered strains contained pCOLA-T7-aroF^wt^-T7-pheA^fbr^ and pCDFD-T7-TcPAL.

^c^Engineered strains contained pCOLA-T7-aroF^wt^-T7-pheA^fbr^, pCDFD-Trc-RgPAL-Trc-4CL, pETD-T7-STS.

^d^Engineered strains contained pCOLA-T7-aroF^wt^-T7-pheA^fbr^, pCDFD-Trc-TcPAL-Trc-4CL, pETD-T7-STS.

Furthermore, by a comparative analysis of pathway intermediate concentrations (Table 1), we found that these two combinations (pCOLA-aroF^wt^-pheA^fbr^, pCDFD-Trc-RgPAL-Trc-4CL or pCDFD-Trc-TcPAL-Trc-4CL, pETD-STS) both led to high accumulation of cinnamic acid. Therefore, results from this metabolite analysis indicated that efficient conversion of cinnamic acid presented the pathway bottleneck, suggesting that subsequent engineering efforts should focus on addressing this obstacle.

### Rational modular design of the overall pathway to remove the pathway bottleneck

To improve the efficiency of this functional pathway, the overall pathway was re-designed as two new modules separated at cinnamic acid rather than previous three modules. Module one comprised a three-gene, upstream pathway to cinnamic acid, which consisted of *aroF* ^wt^, *pheA* ^fbr^ and TcPAL. Module two comprised a two-gene, downstream pathway to pinosylvin, which consisted of 4CL and STS (Fig. 1). Five different plasmids of pCOLADuet-1 (COLA origin), pACYCDuet-1 (p15A origin), pCDFDuet-1 (CDF origin), pETDuet-1 (pBR322 origin) and pRSFDuet-1 (RSF origin) and two promoters of T7 and Trc were used to regulate modular expression. The gene copy numbers of pCOLADuet-1, pACYCDuet-1, pCDFDuet-1, pETDuet-1, and pRSFDuet-1 were assigned as 5, 10, 20, 40 and 100, respectively according to previous reports^15, 19^. The promoter strengths were designated as T7 = 5, Trc = 1^15^.

**Figure 2:**
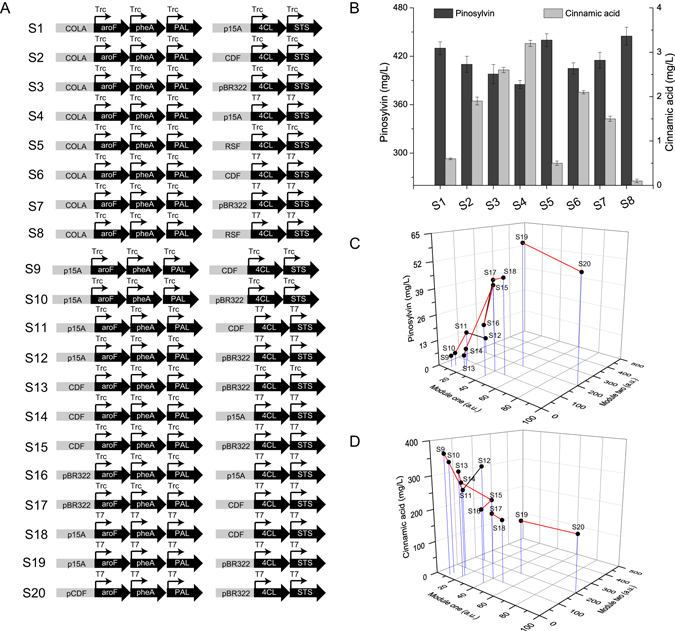
Rational modular design of the overall pathway to remove the pathway bottleneck. (**A**) A series of module one and two expression cassettes were designed at different expression levels. (**B**) The concentrations of pinosylvin were achieved by different expression cassettes (S1–S8). (**C**) Different pinosylvin concentrations were obtained by expression cassettes between S9–S20. (**D**) Different cinnamic acid concentrations were obtained by expression cassettes between S9–S20. COLA: origin of pCOLADuet-1; CDF: origin of pCDFDuet-1; pBR322: origin of pETDuet-1; p15A: origin of pACYCDuet-1; RSF: pRSFDuet-1; Trc: Trc promoter; T7: T7 promoter. S1–S20 demotes strains 1–20 constructed in the study.

At the very start, module two was overexpressed relative to module one to alleviate cinnamic acid accumulation (Fig. 2). In the first round (S1–S8), module one was expressed at a lowest value (COLA × Trc, 5 a.u.), while module two expression increased from a higher value (p15A × Trc, 10 a.u.). It was found that the pinosylvin titer increased from 0.6 mg/L to 3.2 mg/L until an intermediate value (p15A × T7, 50 a.u.) and the titer of intermediate product cinnamic acid decreased from 431 mg/L to 386 mg/L following an opposite trend. This result suggested that the low expression of module one were not suitable for the high expression of module two. Additionally, it was found that use of pRSFDuet-1 in every combination (S5 and S8) led to low titers of pinosylvin and high titers of intermediate cinnamic acid. Furthermore, the final OD600 of these two strains were 2.6 and 2.2, respectively. Hence, it was supposed that the high copy number of pRSFDuet-1 would increase metabolic burden and further lead to negative effect on cell behavior.

Hence, in the second round, module one expression elevated to a higher level (p15A × Trc, 10 a.u.) and module two expression increased from a value of 20 a.u. (CDF × Trc), similar trends were observed and higher pinosylvin production (15.8 mg/L) was obtained. Then in the subsequent rounds of modular engineering, module one expression was set as a value of 20 a.u. (CDF × Trc), 40 a.u. (pBR322 × Trc), 50 a.u. (p15A × T7), 100 a.u. (pCDF × T7), respectively, while module two expression increased from a higher value compared to module one. It was observed that when module one expression elevated to 50 a.u. (p15A × T7), the titer of pinosylvin increased following the increasing expression of module two and highest pinosylvin production (61 mg/L) was obtained with the highest expression of module two (200 a.u., pBR322 × T7).

**Figure 3:**
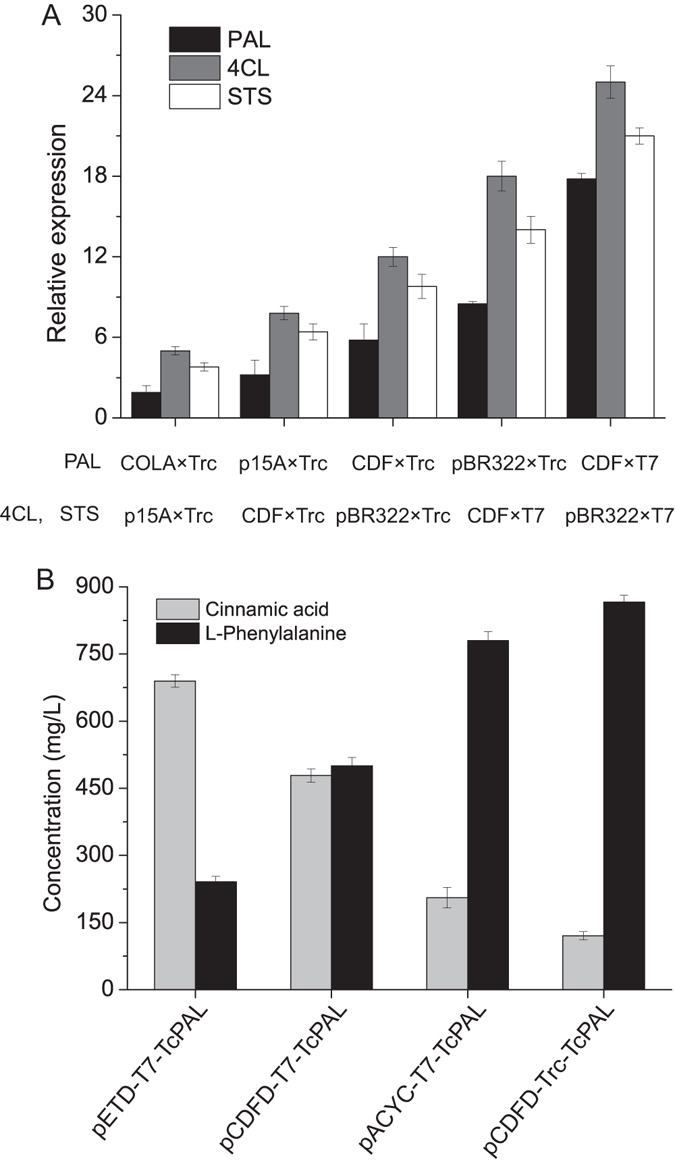
Comparison of the expression level of different plasmids and promoters. (**A**) RT-PCR for transcriptional analysis of heterologous genes. Transcriptional levels of PAL, 4CL and STS were measured by RT-PCR based on mRNA isolated from engineered strains. COLA × Trc, p15A × Trc, CDF × Trc, pBR322 × Trc, CDF × T7, pBR322 × T7 indicated the transcriptional level of 5 a.u., 10 a.u., 20 a.u., 40 a.u., 100 a.u., 200 a.u., respectively. (**B**) Comparison of different PAL *in vivo* activity under different plasmids and promoters. The plasmids of pCDFD-T7-TcPAL, pCDFD-Trc-TcPAL, pACYC-T7-TcPAL and pETD-T7-TcPAL were transformed into BL21 (DE3) strain to compare the *in vivo* TcPAL activity under different plasmids and promoters. These engineered strains were supplemented with 1 g/L L-phenylalanine. Values are calculated after 48 h cultivation in fermentation medium.

In this study, the relative heterologous gene expression strength is calculated based on promoter strength and the plasmid gene copy number. To support this calculation, transcriptional expression levels of TcPAL, 4CL and STS were calculated by qPCR from strains of S1, S9, S13, S17 and S20, as these strains exhibited different gene expression levels. As seen from Fig. 3A, the mRNA transcriptional level directly sustained this calculation method that increasing plasmid gene copy number and promoter strength modulated heterologous gene expression.

To further support this conclusion, the plasmids of pCDFD-T7-TcPAL, pCDFD-Trc-TcPAL, pACYC-T7-TcPAL and pETD-T7-TcPAL were transformed into BL21 (DE3) strain to compare the *in vivo* TcPAL activity under different plasmids and promoters. These engineered strains were supplemented with 1 g/L L-phenylalanine. It was found that pETD-T7-TcPAL (200 a.u.), pCDFD-T7-TcPAL (100 a.u.), pACYC-T7-TcPAL (50 a.u.) and pCDFD-Trc-TcPAL (20 a.u.) obtained 689 mg/L, 478 mg/L, 205 mg/L and 120 mg/L cinnamic acid, respectively (Fig. 3B). This further demonstrated the feasibility of the method described above.

### Optimization of module two by changing 5′ region of mRNA secondary structure

As seen from Fig. 2, in the final round of modular engineering, the titer of pinosylvin increased following the increasing expression of module two and highest pinosylvin production was obtained with the highest expression of module two. That means increasing module two expression would be favorable for pinosylvin production. However, employment of high copy number plasmid pRSFDuet-1 led to high metabolic burden and resulted in negative effect on cell behavior. This genetic recalcitrance restricted the ability to balance the overall pathway. Previously, we demonstrated that reducing the 5′ region secondary structure of the open reading frame of target gene mRNA could function in improving protein expression with no additional metabolic burden^16^. Hence, in this study, this strategy was further developed to optimizing and balancing the heterologous gene expression coding for enzymes of the pinosylvin production pathway.

**Figure 4:**
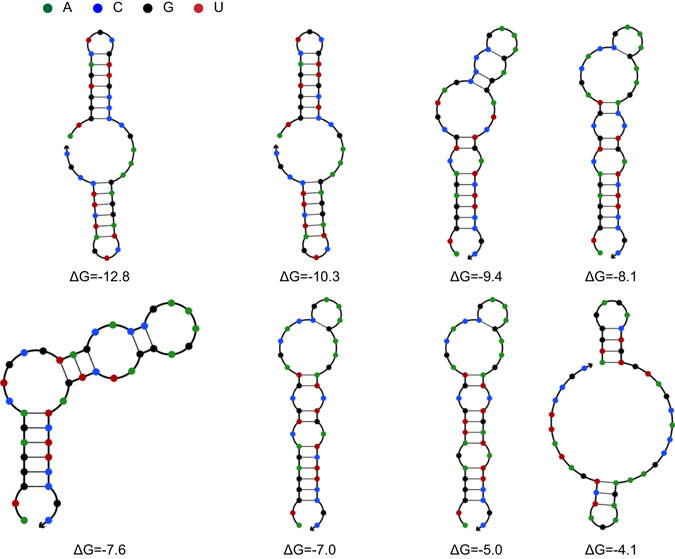
Predicted mRNA secondary structures of the 5′ region of different variant 4CL. Numbers such as −12.8, −10.3 meant ΔG (kcal/mol) for the 5′ region of mRNA secondary structure. The figure exhibited the secondary structure of the region ranged from nt +1 to +42. ΔG of original 4CL was −12.8 kcal/mol.

Firstly, the expression of 4CL was optimized via reducing its 5′ region secondary structure of the open reading frame of mRNA. Based on our previous study^16^, changing synonymous codon usage in the first 14 amino acids of N-terminal protein was performed. For each codon, the first and second positions were kept constant while the third base position mutated randomly. On the basis of translation start site (ATG) to 42 base sequence (+1 to +42), the minimum free energy of folding for the 5′ region of mRNA transcript (ΔG) was calculated by NUPACK software^22^ (Fig. 4).

**Figure 5:**
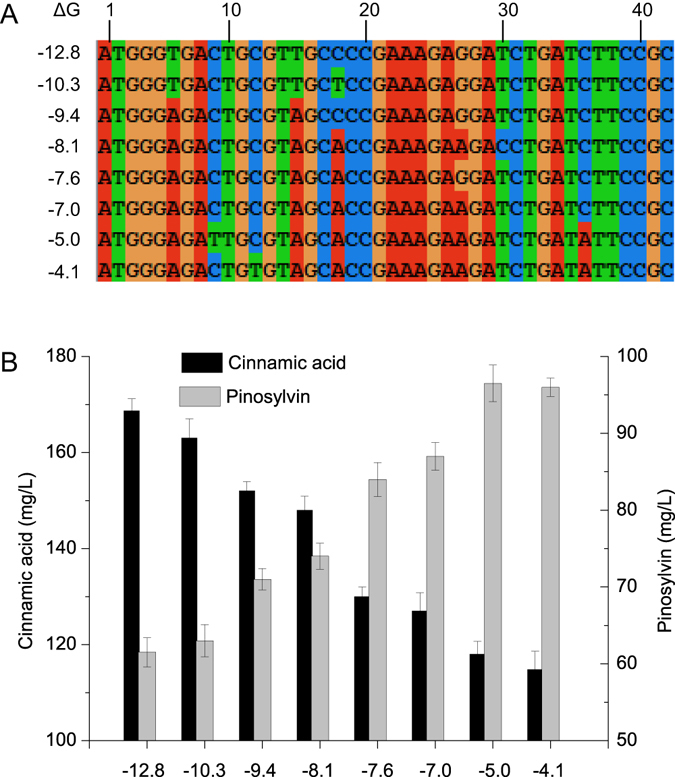
The effect of reducing 5′ region of 4CL mRNA secondary structure on pinosylvin production. (**A**) The alignment of different 4CL variant sequences (+1 to +42). (**B**) Effects of differences in 4CL variant on pinosylvin and cinnamic acid concentrations. Numbers such as −12.8, −10.3 denoted ΔG of 5′ region of 4CL mRNA secondary structure. ΔG of original 4CL was −12.8 kcal/mol.

As seen in Fig. 5, a total of seven variants spanning a range of free energies from −12.8 to −4.1 kcal/mol were chosen. These different variants of 4CL were used to replace the original 4CL in module two to further balance the overall pathway. ΔG of original 4CL was −12.8 kcal/mol. It was found that variants with reduced 5′ region of mRNA secondary structure (larger value of ΔG) increased the pinosylvin production (97 mg/L) until ΔG value of −5.0 kcal/mol. While the concentration of the intermediate product cinnamic acid continuously decreased from 168 to 114 mg/L.

**Figure 6:**
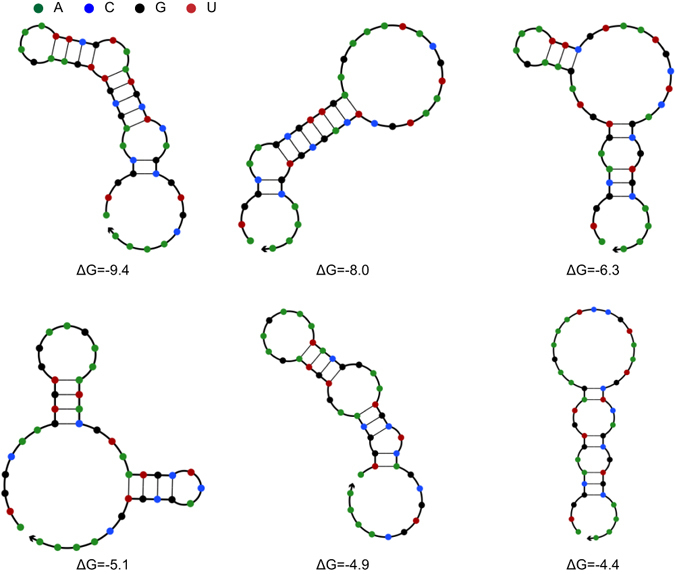
Predicted mRNA secondary structures of the 5′ region of different variant STS. The figure exhibited the secondary structure of the region ranged from nt +1 to nt +42. Numbers such as −9.4, −8.0 meant ΔG (kcal/mol) of the 5′ region of mRNA secondary structure. ΔG of original STS was −9.4 kcal/mol.

**Figure 7:**
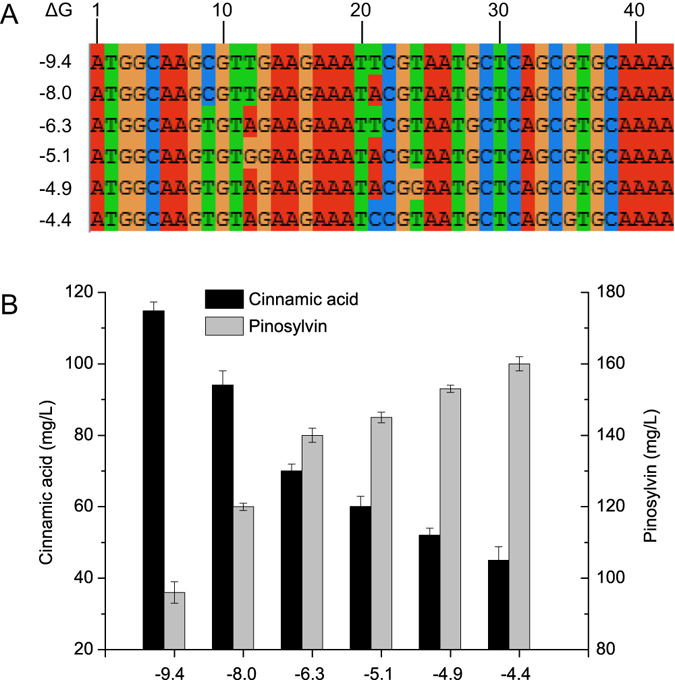
The effect of reducing 5′ region of STS mRNA secondary structure on pinosylvin production. (**A**) The alignment of different STS variant sequences (+1 to +42). (**B**) Effects of differences in STS variant on pinosylvin and cinnamic acid concentrations. Numbers such as −9.4, −8.0 denoted ΔG of 5′ region of STS mRNA secondary structure. ΔG of original STS was −9.4 kcal/mol.

It was observed that continuously reducing the 4CL mRNA secondary structure decreased cinnamic acid accumulation constantly while the pinosylvin production increased until an intermediated value of ΔG. This result meant STS may present as another pathway bottleneck. Hence, six different STS variants spanning a range of free energies from −9.4 to −4.4 kcal/mol were also chosen to replace the original STS (Fig. 6). It was found that variants with reduced 5′ region of mRNA secondary structure increased pinosylvin production from 96 mg/L to 160 mg/L (Fig. 7). It was notably that modifying the expression of STS resulted in a dramatic change of pinosylvin production and the low activity of STS would be another pathway bottleneck.

### Construction of CRISPRi system to enhance malonyl-CoA concentration

To further improve the intracellular availability of the limiting precursor malonyl-CoA, the malonyl-CoA synthesis module (module three) based on CRISPRi was assembled and optimized with other two modules. Based on our previous studies^12, 17^, low repressing efficacy toward genes of *eno*, *adhE fabB* (anti-*eno*, anti-*adhE*, anti-*fabB* sgRNA), medium repressing efficacy toward genes of *sucC* and *fumC* (anti-*sucC*, anti-*fumC* sgRNA) and high repressing efficacy toward genes of *fabF* (anti-*fabF* sgRNA) were conducted in engineered BL21(DE3) strains, because repression of these genes would not alter final biomass significantly while enhancing the intracellular malonyl-CoA level.

**Figure 8:**
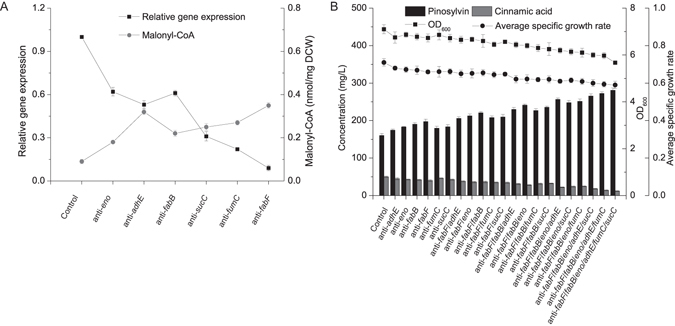
The effect of CRISPRi system on pinosylvin production. (**A**) To verify the efficiency of CRISPRi, the malonyl-CoA concentration and target gene mRNA level were analyzed after repressing. (**B**) Effect of different target gene repression on pinosylvin production were investigated. Control meant strains containing pinosylvin synthetic pathway without repressing. The CRISPRi system silencing different genes were subsequently transformed into the control strain to further improve pinosylvin production. Final OD_600_ values and concentrations of cinnamic acid and pinosylvin were analyzed from engineered strains after 48 h. The average specific growth rate was calculated as (O_2_ × V_2_ − O_1_ × V_1_)/10 × V_2_. O_2_ meant the OD_600_ value at 10 h after induction with IPTG; V_2_ meant fermentation volume after induction (50 mL); O_1_ meant OD_600_ value at the time of induction (1.7); V_1_ meant fermentation volume before induction (25 mL); 10 meant 10 hours of culture time.

In order to verify the repressing efficiency of CRISPRi system, the malonyl-CoA concentration and the changing pattern of target gene mRNA levels from each engineered strain were measured. The target gene mRNA levels from CRISPRi-regulated strains were calculated compared to the control. It was found that the transcriptional levels of *eno*, *adhE*, *fabB*, *sucC*, *fumC*, *fabF* mRNA from CRISPRi-regulated strains decreased by 38.1%, 47.3%, 39.6%, 69.2%, 78.2%, 91.7%, respectively. Silencing of *eno*, *adhE*, *fabB*, *sucC*, *fumC*, *fabF* increased the intracellular malonyl-CoA concentration by 98.4%, 255.9%, 144.4%, 177.8%, 200.7%, 288.9%, respectively (Fig. 8A).

Furthermore, the effect of multiple genetic disturbance on pinosylvin production were explored (Fig. 8B). Different sgRNA sequences involving Trc promoter, complementary region, dCas9-binding hairpin and transcription terminator were inserted into the same plasmid to repress multiple genes. Repressing *adhE*, *eno*, *fabB*, *fabF*, *fumC* and *sucC* increased pinosylvin titer by up to 8.8%, 13.8%, 18.8%, 23.1%, 11.9% and 14.3%, respectively. Then repressing *fabF* with stacking of other genetic interventions were investigated, as the highest pinosylvin titer was obtained when *fabF* was repressed (197 mg/L). It was found that anti-*fabF*/*fabB* sgRNA led to the highest pinosylvin titer (221 mg/L). Similarly, silencing of *fabF* and *fabB* with *adhE*, *eno*, *fumC* and *sucC* were investigated and anti-*fabF*/*fabB*/*eno* sgRNA produced the highest titer (241 mg/L).

Furthermore, we found that anti-*fabF*/*fabB*/*eno* sgRNA associated with anti-*sucC*, anti-*adhE* or anti-*fumC* sgRNA led to higher pinosylvin titer than anti-*fabF*/*fabB*/*eno* sgRNA, and anti-*fabF*/*fabB*/*eno*/*adhE* sgRNA resulted in the highest pinosylvin titer (257 mg/L). Anti-*fabF*/*fabB*/*eno*/*adhE* sgRNA associated with anti-*sucC*, or anti-*fumC* sgRNA also led to higher production titer, and anti-*fabF*/*fabB*/*eno/adhE*/*fumC* sgRNA exhibited the highest titer (272 mg/L). Finally, we demonstrated that anti-*fabF*/*fabB*/*eno/adhE*/*fumC*/*sucC* sgRNA exhibited the highest pinosylvin titer (281 mg/L).

## Discussion

Efficient biosynthesis of pinosylvin from renewable and cheap substrate D-glucose would accelerate the application of pinosylvin as both nutraceutical and pharmaceutical. Previous studies on pinosylvin production have mainly focused on engineering the three-step pathway ranging from L-phenylalanine to pinosylvin^4, 5^. Despite these significant achievements, further improvement in pinosylvin production need investigating metabolic constraints and removing pathway limitations throughout the overall pathway instead of only partial pathway. In this study, by a rational modular design approach, the overall pinosylvin biosynthetic pathway including a total of 11 genes was constructed and arranged into three modules (Fig. 1). These modules were systematically optimized for efficient microbial production and the final engineered strain could produce 281 mg/L pinosylvin. The present work demonstrated that this rational modular design approach would expedite developing robust and efficient microbial cell factory for value-added chemical production.

The ability to more broadly use biological systems for chemical production is always limited by the inefficiency of complex biosynthetic pathways in heterologous hosts^11, 23, 24^. Despite the potential of modular metabolic engineering to revolutionize the field of synthetic biology^15^, the field of synthetic biology still lacks a standard principle for strain optimization. The rational modular design approach described here provides a framework for fine-tuning synthetic pathways, identifying potential pathway bottlenecks and further alleviating them. Initially, different candidate pathway enzymes were screened to construct the overall pathway. Secondly, metabolite profiling indicated that cinnamic acid accumulated in the initial strain (Table 1), the pinosylvin synthetic pathway was then divided at a new node cinnamic acid. These new modules were combinatorially optimized at transcriptional and translational levels, which exhibited a 16-fold increase in pinosylvin titer compared to the initial construct (Fig. 2). It is notably that choosing separating node at cinnamic acid rather than previous cinnamoyl-CoA^16, 17^ lead to a dramatic increase in final production titer. This further highlighted the importance of selecting suitable separating node in modular pathway engineering. The present work reports a rational modular design approach for systematically identifying and removing metabolic bottlenecks.

Compared to the initial modular pathway engineering strategies only employing different plasmids and promoters to fine-tuning pathway efficiency^15, 19, 21^, in this work, we further explored reducing 5′ mRNA secondary structure and synthetic CRISPRi system to modulate pinosylvin biosynthetic pathway and endogenous central metabolism. Based on these combined strategies, our analyses revealed that accumulation of cinnamic acid, low stilbene synthase activity and limited malonyl-CoA availability were the main bottlenecks of the overall pathway and led to engineering efforts that significantly increased pinosylvin titer to 281 mg/L, which represents the highest titer reported to date in microbial production strains. This demonstrated that emerging synthetic devices and strategies in the context of synthetic biology would greatly complement modular pathway engineering to exploit the full potential of cell metabolism^23, 25–27^.

Natural genes often exhibit a bias preference of codon usage and could strongly influence the expression of heterologous genes^28^. In order to improve the expression of heterologous genes, researchers always replace non-optimal codons by optimal ones. However, previous studies find that many organisms are enriched for rare codons at 5′ region of genes and local RNA structure in 5′ region of genes mostly influence expression change^28, 29^. Furthermore, we proved that weakening 5′ region of target gene mRNA secondary structure enhanced gene expression levels dramatically^16^. Here, it was found that increasing module two expression would be favorable for pinosylvin production. However, employment of high copy number plasmid pRSFDuet-1 resulted in negative effect on cell behavior due to the large metabolic burden. Hence, in this study, the strategy was further developed to optimizing module two expression instead of employment of high copy number plasmid. The balance of the overall pathway was finally achieved and the production titer of pinosylvin increased by 1.6-fold (160 vs 62 mg/L). Besides, this strategy described here identified that the low activity of STS was one of the pathway bottlenecks, suggesting a strategy for identifying pathway bottlenecks.

Endogenous central metabolism strongly competes and predominates for energy and carbon sources when synthesizing malonyl-CoA^12, 13^, leaving only a few amounts for pinosylvin production. In one relevant study, researchers demonstrated that addition of fatty acid synthesis inhibitor cerulenin increased production titer significantly^4^. While cerulenin is costly prohibitive for large-scale fermentation process, another study explored using CRISPRi system to repress *fadD* gene and obtained a 1.9-fold increase on production titer^5^. However, it is urgently needed to examine other metabolic engineering targets and further evaluate combinatorial effect of different genetic interventions.

Here, it was found that repression of *eno*, *adhE*, *fabB*, *sucC*, *fumC* and *fabF* could improve the intracellular malonyl-CoA concentration. It was presumed that repression of *eno* would channel carbon flows toward pyruvate (acetyl-CoA precursor), repression of *adhE*, *sucC* and *fumC* would decrease the consumption of acetyl-CoA in TCA cycle or glycolysis and repression of *fabB* and *fabF* would prevent the diversion of malonyl-CoA to the synthesis of fatty acid^12^. Furthermore, the combinatorial effect of various genetic interventions implicated in central metabolic pathways was explored and the best combination was obtained (Fig. 8). Our study demonstrated that CRISPRi system coupled with modular pathway engineering strategy are powerful tools with which to expand methods and strategies for systematic engineering of industrially important microorganisms.

## Materials and Methods

### General techniques

Luria broth (LB) and *E. coli* JM109 were employed for plasmid construction, MOPS minimal medium^30^ with additional 5 g/L glucose and 4 g/L NH_4_Cl and *E. coli* BL21 (DE3) were employed to express heterologous genes. Ampicillin (100 μg/mL), kanamycin (40 μg/mL), chloramphenicol (20 μg/mL), and streptomycin (40 μg/mL) were supplemented to maintain the existing of the compatible vectors pETDuet-1, pRSFDuet-1, pACYCDuet-1, pCDFDuet-1, pRSFDuet-1, and pACYCDuet-1 (Novagen, Darmstadt, Germany). Pinosylvin was purchased from Sigma-Aldrich (P56297). DNA ligase and restriction enzymes were purchased from Novagen (Darmstadt, Germany). PAL from *Rhodotorula glutinis* (RgPAL)^31^, PAL from *Trichosporon cutaneum* (TcPAL)^20^, 4CL from *Petroselinum crispum*, STS from *Vitis vinifera* ^6^ were codon-optimized for *E. coli* expression (http://www.jcat.de/), synthesized by GenScript (Nanjing, China). The sequences of all synthesized genes are showed in the Supplementary Material. A UV/vis spectrophotometer (UVmini-1240, Shimadzu, Kyoto, Japan) was used to measure absorbance at 600 nm (OD_600_).

### Synthetic pathway construction

Colony PCR and Sanger sequencing were employed to confirm all constructed plasmids. Primers and plasmids present in this study are showed in Tables 2 and 3, respectively.

**Table 2:** Nucleotide sequences of primers.

Oligonucleotides	Sequences, 5′-3′^a^
Pf_Trc (*Eco*NI)	**CCTGCATTAGG**CCGACATCATAAC
Pf_Trc (*Pfo*I)	**TCCGGGA**CCGACATCATAAC
Pr_Trc (*Fse*I)	**GGCCGGCC**CAACAGATAAAACGAAAGGCC
Pf_TcPAL (*Nco*I)	CATG**CCATGG**GCATGTTTATTGAAACCAACGTGGCAA
Pr_TcPAL (*Eco*RI)	CCG**GAATTC**TTAAAACATTTTACCCACTGCACCC
Pr_TcPAL (*Xho*I)	CCG**CTCGAG**TTAAAACATTTTACCCACTGCACCC
Pf_4CL (*Nco*I)	CATG**CCATGG**GTGACTGCGTTGCCCCG
Pr_4CL (*Hin*dIII)	C**AAGCTT**TTACTTCGGCAGGTCGCCGCTC
Pr_4CL (*Eco*RI)	CCG**GAATTC**TTACTTCGGCAGGTCGCCGCTC
Pf_Ptrc4CL (*Eco*RI)	CCG**GAATTC**CCGACATCATAACGGTTCTGG
Pr_Ptrc4CL (*Hin*dIII)	CCC**AAGCTT**CAACAGATAAAACGAAAGGCCC
Pf_AroF (*Nco*I)	CATG**CCATGG**GCATGCAAAAAGACGCGCTGAAT
Pr_AroF (*Hin*dIII)	CCC**AAGCTT**TTAAGCCACGCGAGCCGT
Pf_PtrcAroF (*Eco*NI)	**CCTGCATTAGG**CCGACATCATAACGGTTCTGG
Pf_PtrcAroF (*pfo*I)	**TCCGGGA**CCGACATCATAACGGTTCTGG
Pr_PtrcAroF (*Bam*HI)	CGC**GGATCC**CAACAGATAAAACGAAAGGCCC
Pf_PheA (*Nco*I)	CATG**CCATGG**GCATGACATCGGAAAACC
Pr_PheA (*Hin*dIII)	CCC**AAGCTT**TCAGGTTGGATCAACAG
Pr_PheA (*Xho*I)	CCG**CTCGAG**TCAGGTTGGATCAACAG
Pf_PtrcPheA (*Bam*HI)	CGC**GGATCC**CCGACATCATAACGGTTCTGG
Pr_PtrcPheA (*Nde*I)	GGAATTC**CATATG**CAACAGATAAAACGAAAGGCCC
Pf_PtrcTcPAL (*Nde*I)	GGAATTC**CATATG**CCGACATCATAACGGTTCTGG
Pr_PtrcTcPAL (*Xho*I)	CCG**CTCGAG**TTAAAACATTTTACCCACTGCACCC
Pf_Pt7PheA (*Hin*dIII)	C**AAGCTT**GGATCTCGACGCTCTCCCT
Pr_Pt7PheA (*Nde*I)	GGAATTC**CATATG**GCTAGTTATTGCTCAGCGG
Pf_Pt7TcPAL (*Nde*I)	GGAATTC**CATATG**GGATCTCGACGCTCTCCCT
Pr_Pt7TcPAL (*Avr*II)	**CCTAGG**GCTAGTTATTGCTCAGCGG
Pf_STS (*Nco*I)	**CCATGG**CAAGCGTTGAAGAAAT
Pr_STS (*Hin*dIII)	CCC**AAGCTT**TCAGTTCGTAACCATCGGAAT
Pf_PtrcSTS (*Eco*RI)	CCG**GAATTC**CCGACATCATAACGGTTCTGG
Pr_PtrcSTS (*Hin*dIII)	CCC**AAGCTT**CAACAGATAAAACGAAAGGCCC
Pf_STS (*Nde*I)	GGAATTC**CATATG**GCAAGCGTTGAAGAAAT
Pr_STS (*Avr*II)	**CCTAGG**TCAGTTCGTAACCATCGGAAT
Pf_sgRNA (*Bam*HI)	CGC**GGATCC**TGTACACTGCAGGTCGTAAATCAC
Pr_sgRNA (*Eco*RI)	CCG**GAATTC**AAAAAAGCACCGACTCGGTG
Pf_sgRNA (*Eco*RI)	CCG**GAATTC**TGTACACTGCAGGTCGTAAATCAC
Pr_sgRNA (*Hin*dIII)	CCC**AAGCTT**AAAAAAGCACCGACTCGGTG
Pf_sgRNA (*Hin*dIII)	CCC**AAGCTT**TGTACACTGCAGGTCGTAAATCAC
Pr_sgRNA (*Nde*I)	GGAATTC**CATATG**AAAAAAGCACCGACTCGGTG
Pf_sgRNA (*Nde*I)	GGAATTC**CATATG**TGTACACTGCAGGTCGTAAATCAC
Pr_sgRNA (*Bgl*II)	GA**AGATCT**AAAAAAGCACCGACTCGGTG
Pf_sgRNA (*Bgl*II)	GA**AGATCT**TGTACACTGCAGGTCGTAAATCAC
Pr_sgRNA (*Kpn*I)	GG**GGTACC**AAAAAAGCACCGACTCGGTG
Pf_4CL (−10.3)	ATGGGTGACTGCGTTGCTCCGAAAGAGGATCTGATCTTCCGC
Pr_4CL (−10.3)	GCGGAAGATCAGATCCTCTTTCGGAGCAACGCAGTCACCCAT
Pf_4CL (−9.4)	ATGGGAGACTGCGTAGCCCCGAAAGAGGATCTGATCTTCCGC
Pr_4CL (−9.4)	GCGGAAGATCAGATCCTCTTTCGGGGCTACGCAGTCTCCCAT
Pf_4CL (−8.1)	ATGGGAGACTGCGTAGCACCGAAAGAAGACCTGATCTTCCGC
Pr_4CL (−8.1)	GCGGAAGATCAGGTCTTCTTTCGGTGCTACGCAGTCTCCCAT
Pf_4CL (−7.6)	ATGGGAGACTGCGTAGCACCGAAAGAGGATCTGATCTTCCGC
Pr_4CL (−7.6)	GCGGAAGATCAGATCCTCTTTCGGTGCTACGCAGTCTCCCAT
Pf_4CL (−7.0)	ATGGGAGACTGCGTAGCACCGAAAGAAGATCTGATCTTCCGC
Pr_4CL (−7.0)	GCGGAAGATCAGATCTTCTTTCGGTGCTACGCAGTCTCCCAT
Pf_4CL (−5.0)	ATGGGAGATTGCGTAGCACCGAAAGAAGATCTGATATTCCGC
Pr_4CL (−5.0)	GCGGAATATCAGATCTTCTTTCGGTGCTACGCAATCTCCCAT
Pf_4CL (−4.1)	ATGGGAGACTGTGTAGCACCGAAAGAAGATCTGATATTCCGC
Pr_4CL (−4.1)	GCGGAATATCAGATCTTCTTTCGGTGCTACACAGTCTCCCAT
Pf_STS (−8.0)	ATGGCAAGCGTTGAAGAAATACGTAATGCTCAGCGTGCAAAA
Pr_STS (−8.0)	TTTTGCACGCTGAGCATTACGTATTTCTTCAACGCTTGCCAT
Pf_STS (−6.3)	ATGGCAAGTGTAGAAGAAATTCGTAATGCTCAGCGTGCAAAA
Pr_STS (−6.3)	TTTTGCACGCTGAGCATTACGAATTTCTTCTACACTTGCCAT
Pf_STS (−5.1)	ATGGCAAGTGTGGAAGAAATACGTAATGCTCAGCGTGCAAAA
Pr_STS (−5.1)	TTTTGCACGCTGAGCATTACGTATTTCTTCCACACTTGCCAT
Pf_STS (−4.9)	ATGGCAAGTGTAGAAGAAATACGGAATGCTCAGCGTGCAAAA
Pr_STS (−4.9)	TTTTGCACGCTGAGCATTCCGTATTTCTTCTACACTTGCCAT
Pf_STS (−4.4)	ATGGCAAGTGTAGAAGAAATACGTAATGCTCAGCGTGCAAAA
Pr_STS (−4.4)	TTTTGCACGCTGAGCATTACGTATTTCTTCTACACTTGCCAT
Pf_qPAL	CGTGGTACCATCAGTGCATC
Pr_qPAL	ACTGCCGTACCGTTAACCAG
Pf_q4CL	CGGCGAAACCTTTACCTACA
Pr_q4CL	AGAATGGGTTCGCCATAGTG
Pf_qSTS	GTAATGCTCAGCGTGCAAAA
Pr_qSTS	ATGTTCGGGTGTTCTTCCAG
Pf_qeno	GGCGAAACTGAAGACGCTAC
Pr_qeno	GACCGTTGTACGGTGCTTTT
Pf_qadhE	CGAAGACGCGGTAGAAAAAG
Pr_qadhE	AACCCAGAGTCAGGGAAGGT
Pf_qfumC	CTGCGGAATTGGTGAAATCT
Pr_qfumC	TTGCAGGAAATTGTGGATCA
Pf_qsucC	GGTTAACATCTTCGGCGGTA
Pr_qsucC	CTGCATCCGTCAGACCTTTT
Pf_qfabB	AGATCCAACTGGGCAAACAG
Pr_qfabB	CACGGTGAGCGTCGTAAGTA
Pf_qfabF	GACTGGGCATGTTGTCTCCT
Pr_qfabF	GCCAGCGACAATTCCATATT
Pf_16S	TTGCTCATTGACGTTACCCG
Pr_16S	GTTGCACCACAGATG AAACG

^a^Bold and underlined letters are restriction enzyme cut sites.

**Table 3:** Plasmids used in this study.

Plasmids	Description	Source or reference
pETDuet-1	Double T7 promoters, pBR322 ori, Amp^R^	Novagen
pCDFDuet-1	Double T7 promoters, CloDF13 ori, Sm^R^	Novagen
pACYCDuet-1	Double T7 promoters, P15A ori, Cm^R^	Novagen
pRSFDuet-1	Double T7 promoters, RSF ori, Kn^R^	Novagen
pCOLADuet-1	Double T7 promoters, COLA ori, Kn^R^	Novagen
pCDFD-Trc	T7 promoter was replaced by Trc promoter	This study
pRSFD-Trc	T7 promoter was replaced by Trc promoter	This study
pACYC-Trc	T7 promoter was replaced by Trc promoter	This study
pETD-Trc	T7 promoter was replaced by Trc promoter	This study
pCDFD-T7-TcPAL	pCDFDuet-1 carrying TcPAL under T7 promoter	This study
pCDFD-T7-RgPAL	pCDFDuet-1 carrying RgPAL under T7 promoter	This study
pCDFD-Trc-TcPAL	pCDFDuet-1 carrying TcPAL under Trc promoter	This study
pACYC-T7-TcPAL	pACYCDuet-1 carrying TcPAL under T7 promoter	This study
pCOLA-T7-TcPAL	pCOLADuet-1 carrying TcPAL under T7 promoter	This study
pETD-T7-TcPAL	pETDuet-1 carrying TcPAL under T7 promoter	This study
pCDFD-Trc-4CL	pCDFDuet-1 carrying 4CL under Trc promoter	This study
pCDF-Trc-TcPAL-Trc-4CL	pCDFDuet-1 Carrying TcPAL and 4CL under Trc promoter	This study
pCOLA-T7-aroF^wt^-T7-pheA^fbr^	pCOLADuet-1 carrying *aroF* ^wt^ and *pheA* ^fbr^	16
pCDFD-Trc-aroF^wt^	pCDFDuet-1 carrying 4CL under Trc promoter	This study
pCOLA-Trc-aroF^wt^-Trc-pheA^fbr^	pCOLADuet-1 carrying *aroF* ^wt^ and *pheA* ^fbr^ under Trc promoter	This study
pCOLA-Trc-aroF^wt^-Trc-pheA^fbr^-Trc-TcPAL	pCOLADuet-1 carrying *aroF* ^wt^, *pheA* ^fbr^ and TcPAL under Trc promoter	This study
pACYC-Trc-aroF^wt^-Trc-pheA^fbr^-Trc-TcPAL	pACYCDuet-1 carrying *aroF* ^wt^, *pheA* ^fbr^ and TcPAL under Trc promoter	This study
pCDFD-Trc-aroF^wt^-Trc-pheA^fbr^-Trc-TcPAL	pCDFDuet-1 carrying *aroF* ^wt^, *pheA* ^fbr^ and TcPAL under Trc promoter	This study
pETD-Trc-aroF^wt^-Trc-pheA^fbr^-Trc-TcPAL	pETDuet-1 carrying *aroF* ^wt^, *pheA* ^fbr^ and TcPAL under Trc promoter	This study
pCDFD-T7-aroF^wt^-T7-pheA^fbr^-T7-TcPAL	pCDFDuet-1 carrying *aroF* ^wt^, *pheA* ^fbr^ and TcPAL under T7 promoter	This study
pACYC-T7-aroF^wt^-T7-pheA^fbr^-T7-TcPAL	pACYCDuet-1 carrying *aroF* ^wt^, *pheA* ^fbr^ and TcPAL under T7 promoter	This study
pCDFD-Trc-4CL-Trc-STS	pCDFDuet-1 carrying 4CL and STS under Trc promoter	This study
pETD-Trc-4CL-Trc-STS	pETDuet-1 carrying 4CL and STS under Trc promoter	This study
pACYC-Trc-4CL-Trc-STS	pACYCDuet-1 carrying 4CL and STS under Trc promoter	This study
pRSFD-Trc-4CL-Trc-STS	pRSFDuet-1 carrying 4CL and STS under Trc promoter	This study
pCDFD-T7-4CL-T7-STS	pCDFDuet-1 carrying 4CL and STS under T7 promoter	This study
pACYC-T7-4CL-T7-STS	pACYCDuet-1 carrying 4CL and STS under T7 promoter	This study
pRSFD-T7-4CL-T7-STS	pRSFDuet-1 carrying 4CL and STS under T7 promoter	This study
pET-T7-4CL-T7-STS	pETDuet-1 carrying 4CL and STS under T7 promoter	This study
pETD-T7-4CL(*N*)-T7-STS^a^	T7 promoter was replaced by Trc promoter	This study
pETD-T7-4CL(−5.0)-T7-STS(M)^b^	pCDFDuet-1 carrying STS	This study

^a^ *N* = −10.3, −9.4, −8.1, −7.6, −7.0, −5.0, −4.1.

^b^ *M* = −8.0, −6.3, −5.1, −4.9, −4.4.

Primers Pf_Trc (*Pfo*I) and Pr_Trc (*Fse*I) were employed to clone the Trc promoter, multi-cloning sites and corresponding terminator from pTrcHis2B to the sites of *Pfo*I/*Fse*I in pCDFDuet-1, pRSFDuet-1, respectively. This resulted in pCDFD-Trc, pRSFD-Trc. Primers Pf_Trc (*Eco*NI) and Pr_Trc (*Fse*I) were used to clone the Trc promoter, multi-cloning sites and *rrnB* terminator from pTrcHis2B to the sites of *Eco*NI/*Fse*I in pACYCDuet-1, pETDuet-1, resulted in pACYC-Trc, pETD-Trc. PAL from *Trichosporon cutaneum* (TcPAL) was independently cloned from pUC57-TcPAL (synthesized by GenScript, Nanjing, China) into pCDFD-Trc using primers Pf_TcPAL (*Nco*I) and Pr_TcPAL (*Eco*RI) with enzymes *Nco*I and *Eco*RI, which resulted in pCDFD-Trc-TcPAL. 4CL was cloned from pUC57-4CL (synthesized by GenScript, Nanjing, China) into pCDFD-Trc by primers Pf_4CL (*Nco*I) and Pr_4CL (*Hin*dIII) and the enzymes *Nco*I and *Hin*dIII, resulted in pCDFD-Trc-4CL. pCDFD-Trc-TcPAL-Trc-4CL was constructed by amplifying Trc promoter and 4CL (pTrc-4CL region) with primers Pf_Ptrc4CL (*Eco*RI) and Pr_Ptrc4CL (*Hin*dIII) and cloning into pCDFD-Trc-TcPAL with *Eco*RI and *Hin*dIII.

*aroF* ^wt^ was cloned from pCOLA-T7-aroF^wt^-T7-pheA^fbr^ ^16^ into pCDFD-Trc using primers Pf_AroF (*Nco*I) and Pr_AroF (*Hin*dIII) with enzymes *Nco*I and *Hin*dIII, which resulted in pCDFD-Trc-aroF^wt^. pTrc-aroF^wt^ region of Trc promoter and *aroF* ^wt^ was cloned with primers Pf_PtrcAroF (*Pfo*I) and Pr_PtrcAroF (*Bam*HI) and cloned into pCOLADuet-1 with *Pfo*I and *Bam*HI, resulted in pCOLA-Trc-aroF^wt^. *pheA* ^fbr^ was cloned from pCOLA-T7-aroF^wt^-T7-pheA^fbr^ into pCDFD-Trc by primers Pf_PheA (*Nco*I) and Pr_PheA (*Hin*dIII) and the enzymes *Nco*I and *Hin*dIII, resulted in pCDFD-Trc-pheA^fbr^. pCOLA-Trc-aroF^wt^-Trc-pheA^fbr^ was constructed by amplifying the pTrc-pheA^fbr^ region including Trc promoter and *pheA* ^fbr^ from pCDFD-Trc-pheA^fbr^ with primers Pf_PtrcPheA (*Bam*HI) and Pr_PtrcPheA (*Nde*I) and cloning into pCOLA-Trc-aroF^wt^ with *Bam*HI and *Nde*I. To construct pCOLA-Trc-aroF^wt^-Trc-pheA^fbr^-Trc-TcPAL, the pTrc-TcPAL region including Trc promoter and TcPAL was amplifying from pCDFD-Trc-TcPAL with primers Pf_PtrcTcPAL (*Nde*I) and Pr_PtrcTcPAL (*Xho*I) and cloning into pCOLA-Trc-aroF^wt^-Trc-pheA^fbr^ with *Nde*I and *Xho*I. pACYC-Trc-aroF^wt^-Trc-pheA^fbr^-Trc-TcPAL, pCDFD-Trc-aroF^wt^-Trc-pheA^fbr^-Trc-TcPAL and pETD-Trc-aroF^wt^-Trc-pheA^fbr^-Trc-TcPAL were constructed through amplifying the pTrc-aroF^wt^-pTrc-pheA^fbr^-pTrc-TcPAL region with primers Pf_PtrcAroF (*Eco*NI) and Pr_PtrcTcPAL (*Xho*I) and cloning into pACYCDuet-1, pCDFDuet-1, pETDuet-1 with *Eco*NI and *Xho*I, respectively.

*aroF* ^wt^ was cloned from pCOLA-T7-aroF^wt^-T7-pheA^fbr^ into pCDFDuet-1 using primers Pf_AroF (*Nco*I) and Pr_AroF (*Hin*dIII) with enzymes *Nco*I and *Hin*dIII, which resulted in pCDFD-T7-aroF^wt^. *pheA* ^fbr^ was cloned from pCOLA-T7-aroF^wt^-T7-pheA^fbr^ into pCDFDuet-1 by primers Pf_PheA (*Nco*I) and Pr_PheA (*Xho*I) and the enzymes *Nco*I and *Xho*I, resulted in pCDFD-T7-pheA^fbr^. pCDFD-T7-aroF^wt^-T7-pheA^fbr^ was constructed by amplifying the pT7-pheA^fbr^ region including T7 promoter and *pheA* ^fbr^ from pCDFD-T7-pheA^fbr^ with primers Pf_Pt7PheA (*Hin*dIII) and Pr_Pt7PheA (*Nde*I) and cloning into pCDFD-T7-aroF^wt^ with *Hin*dIII and *Nde*I. TcPAL was cloned from pUC57-TcPAL into pCDFDuet-1 by primers Pf_TcPAL (*Nco*I) and Pr_TcPAL (*Xho*I) and the enzymes *Nco*I and *Xho*I, resulted in pCDFD-T7-TcPAL. To construct pCDFD-T7-aroF^wt^-T7-pheA^fbr^-T7-TcPAL, the pT7-TcPAL region including T7 promoter and TcPAL was amplified from pCDFD-T7-TcPAL with primers Pf_Pt7TcPAL (*Nde*I) and Pr_Pt7TcPAL (*Avr*II) and cloning into pCDFD-T7-aroF^wt^-T7-pheA^fbr^ with *Nde*I and *Avr*II. pACYC-T7-aroF^wt^-T7-pheA^fbr^-T7-TcPAL was constructed through the digestion of pCDFD-T7-aroF^wt^-T7-pheA^fbr^-T7-TcPAL and pACYCDuet-1 with enzymes *Eco*NI and *Avr*II, followed by ligation of the appropriate fragments.

4CL was independently cloned into pCDFD-Trc, pETD-Trc, pRSFD-Trc and pACYC-Trc from pUC57-4CL (GenScript, Nanjing, China) via primers Pf_4CL (*Nco*I) and Pr_4CL (*Eco*RI) with the restriction enzymes *Nco*I and *Eco*RI, respectively. This resulted in pCDFD-Trc-4CL, pETD-Trc-4CL, pRSFD-Trc-4CL, pACYC-Trc-4CL. STS was independently cloned into pCDFD-Trc from pUC57-STS (GenScript, Nanjing, China) via primers Pf_STS (*Nco*I) and Pr_STS (*Hin*dIII) with the restriction enzymes *Nco*I and *Hin*dIII, resulted in pCDFD-Trc-STS. The pTrc-STS region including Trc promoter and STS was amplified from pCDFD-Trc-STS with primers Pf_PtrcSTS (*Eco*RI) and Pr_PtrcSTS (*Hin*dIII) and cloned into pCDFD-Trc-4CL, pETD-Trc-4CL, pACYC-Trc-4CL, pRSFD-Trc-4CL with *Eco*RI and *Hin*dIII to construct pCDFD-Trc-4CL-Trc-STS, pETD-Trc-4CL-Trc-STS, pACYC-Trc-4CL-Trc-STS and pRSFD-Trc-4CL-Trc-STS.

4CL from pUC57-4CL was cloned from pUC57-4CL via primers Pf_4CL (*Nco*I) and Pr_4CL (*Hin*dIII) and the resulting product was inserted into the *Nco*I/*Hin*dIII sites of pETDuet-1, resulted in pETD-T7-4CL. pET-T7-4CL-T7-STS was constructed by amplifying STS from pUC57-STS via primers Pf_STS (*Nde*I) and Pr_STS (*Avr*II) and inserting resulting products into the *Nde*I/*Avr*II sites of pETD-T7-4CL. pCDFD-T7-4CL-T7-STS, pACYC-T7-4CL-T7-STS and pRSFD-T7-4CL-T7-STS were constructed through the digestion of pETD-T7-4CL-T7-STS and pCDFDuet-1, pACYCDuet-1, pRSFDuet-1 with enzymes *Nco*I and *Avr*II, followed by ligating the appropriate fragments.

### Construction of different variant 4CL and STS

pETD-T7-4CL(*N*)-T7-STS was constructed via QuikChange II XL Site-Directed Mutagenesis Kit (Agilent Technologies, Santa Clara, CA) with primers Pf_4CL(*N*) and Pr_4CL(*N*) (*N* = −10.3, −9.4, −8.1, −7.6, −7.0, −5.0, −4.1 kcal/mol, values mean the free energy of every 4CL secondary structure) and the template pETD-T7-4CL(*N*)-T7-STS. pETD-T7-4CL(−5.0)-T7-STS(M) was constructed via QuikChange II XL Site-Directed Mutagenesis Kit (Agilent Technologies, Santa Clara, CA) and primers Pf_STS(*M*) and Pr_STS(*M*) (*M* = −8.0, −6.3, −5.1, −4.9, −4.4 kcal/mol, values mean the free energy of every STS secondary structure).

### Construction of CRISPRi system to repress target genes

To conduct the CRISPRi platform in *E. coli*, dCas9 protein (catalytically invalid Cas9 mutant) and the sgRNA including Trc promoter, complementary sequence, dCas9-binding hairpin and corresponding terminator were overexpressed^12, 17, 32^. Different sgRNA cassettes repressing single gene *adhE*, *eno*, *fabB*, *fabF*, *fumC* and *sucC* were constructed by site-directed mutagenesis according to TaKaRa MutanBEST Kit (Takara Biotechnology, Dalian, China) as described before^12, 17^.

Different sgRNA cassettes including promoter, dCas9-binding region and transcription terminator toward each target gene were cloned into one plasmid to silence multiple genes. Primers Pf_sgRNA(*Bam*HI)/Pr_sgRNA(*Eco*RI) were employed to clone anti-*adhE*, anti-*eno*, anti-*fabB*, anti-*fumC* and anti-*sucC* sgRNA from individual sgRNA cassette into the *Bam*HI/*Eco*RI positions of pCOLA-fabF(high), led to pCOLA-fabF(high)/adhE(low), pCOLA-fabF(high)/eno(low), pCOLA-fabF(high)/fabB(low), pCOLA-fabF(high)/fumC(medium) and pCOLA-fabF(high)/sucC(medium). Primers Pf_sgRNA(*Eco*RI)/Pr_sgRNA(*Hin*dIII) were employed to clone anti-*adhE*, anti-*eno*, anti-*fumC* and anti-*sucC* sgRNA from individual sgRNA cassette into the *Eco*RI/*Hin*dIII positions of pCOLA-fabF(high)/fabB(low), led to pCOLA-fabF(high)/fabB(low)/adhE(low), pCOLA-fabF(high)/fabB(low)/eno(low), pCOLA-fabF(high)/fabB(low)/fumC(medium) and pCOLA-fabF(high)/fabB(low)/sucC(medium). Primers Pf_sgRNA(*Hin*dIII)/Pr_sgRNA(*Nde*I) were employed to clone anti-*adhE*, anti-*sucC* and anti-*fumC* sgRNA from individual sgRNA cassette into the *Hin*dIII/*Nde*I positions of pCOLA-fabF(high)/fabB(low)/eno(low), led to pCOLA-fabF(high)/fabB(low)/eno(low)/adhE(low), pCOLA-fabF(high)/fabB(low)/eno(low)/sucC(medium) and pCOLA-fabF(high)/fabB(low)/eno(low)/fumC(medium). Primers Pf_sgRNA(*Nde*I)/Pr_sgRNA(*Bgl*II) were utilized to clone anti-*sucC* and anti-*fumC* sgRNA into the *Nde*I/*Bgl*II positions of pCOLA-fabF(high)/fabB(low)/eno(low)/adhE(low), led to pCOLA-fabF(high)/fabB(low)/eno(low)/adhE(low)/sucC(medium) and pCOLA-fabF(high)/fabB(low)/eno(low)/adhE(low)/fumC(medium). Primers Pf_sgRNA(*Bgl*II)/Pr_sgRNA(*Kpn*I) were utilized to clone anti-*sucC* sgRNA into the *Bgl*II/*Kpn*I positions of pCOLA-fabF(high)/fabB(low)/eno(low)/adhE(low)/fumC(medium), led to pCOLA-fabF(high)/fabB(low)/eno(low)/adhE(low)/fumC(medium)/sucC(medium).

### Culture conditions

For microbial production of pinosylvin, cells were firstly cultured in 25 mL of MOPS minimal medium^30^ with additional 5 g/L D-glucose and 4 g/L NH_4_Cl. This was conducted in 500 mL shake flasks with 220 rpm orbital shaking at 37 °C. When OD_600_ of the culture reached 1.7, another 25 mL of fresh medium were provided. Cells were then cultivated at 30 °C supplemented with 1 mM IPTG. The pinosylvin and cinnamic acid concentrations and the final OD_600_ values were calculated after 48 h.

### RNA preparation and qPCR

Recombinant cells were collected when cells reached at the stationary phase and subsequently frozen by liquid nitrogen. Total RNA was purified via RNAprep Pure Kit (TIANGEN, Beijing, China). RNeasy Mini Kit ((Takara, Dalian, China) was utilized to remove genomic DNA. NanoDrop ND-1000 Spectrophotometer was utilized to measure RNA quality and quantification (NanoDrop Technologies, Wilmington, DE). The SuperScriptTM III First-Strand Synthesis System (Invitrogen) and total RNA were used to synthesize cDNA^33^.

Primers Pf_qPAL/Pr_qPAL, Pf_q4CL/Pr_q4CL, Pf_qSTS/Pr_qSTS, Pf_qeno/Pr_qeno, Pf_qadhE/Pr_qadhE, Pf_qfumC/Pr_qfumC, Pf_qsucC/Pr_qsucC, Pf_qfabB/Pr_qfabB and Pf_qfabF/Pr_qfabF were used to amplify PAL, 4CL, STS, *eno*, *adhE*, *fumC*, *sucC*, *fabB* and *fabF* with cDNA as templates. Primers were first designed by Primer 3 software (http://bioinfo.ut.ee/primer3-0.4.0/). The specificity of primers were verified by running the resulting amplifying DNA products in agarose gels. RT-PCR assays were conducted by SYBR green PCR Master Mix (Takara, Dalian, China) on a LightCycler 480 II thermal cycler system (Roche, Mannheim, Germany)^34^. Five dilutions of the obtaining cDNAs were used to verify the efficiencies of RT-PCR and a negative control were conducting without cDNA added to confirm purity of the sample. LightCycler software was used to calculate threshold cycle (*C*_*T*_) values based on fluorescent values. qPCR values were normalized by the housekeeping *rrsD* gene via Pf_16S and Pr_16S^35^. Relative expression of each gene was estimated according to comparative 2^−ΔΔCt^ method^36^. Each experiment was performed in triplicate and mean values were utilized for analysis.

### Analytical methods

Triplicate cultures were conducted and their deviation is represented by an error bar for each experiment. Error bars show s.d. with 95% confidence interval (Cl). To analyze pinosylvin and cinnamic acid production, 1 mL of supernatant was mixed with 1 mL of ethyl acetate after separating cells through centrifugation (5000 *g*, 15 min, 4 °C). Vortexing and centrifugation (5000 *g*, 15 min, 4 °C) were conducted, followed by evaporating the top organic layer to dryness. 1 mL of methanol was used to re-solubilize the remaining residue. Agilent 1100 series HPLC instrument equipped with a reverse-phase Gemini NX-C18 column (5 × 110 mm) was utilized for analyzing samples. The column was maintained at 25 °C. Pinosylvin and cinnamic acid were analyzed with an acetonitrile/1% acetic acid gradient: 5% acetonitrile (vol/vol) for 5 min, 5 to 80% acetonitrile for 13 min, 80% acetonitrile for 5 min, 80 to 5% acetonitrile for 4 min. The flow rate was set as 1.0 mL/min.

For L-phenylalanine analysis, a 0.45 µm cellulose membrane (Sangon Biotech, Shanghai, China) was used to filter culture supernatants for HPLC analysis and a Supelco Discovery C18 column (250 × 4.6 mm, 5 μm) (Sigma, St. Louis, MO) was employed. Mobile phase was 0.2% trifluoroacetic acid in 40% methanol at 0.5 ml/min. L-phenylalanine was detected by photodiode array at 220 nm and other metabolites were at 280 nm. For malonyl-CoA concentration quantifying, 1 mL of cell culture was chilled and centrifuged (10000 *g*, 4 °C) for 15 min. 1 mL of 6% perchloric acid was used to re-suspend cell pellet to facilitate cell lysis. 300 μL of 3 M potassium carbonate was utilized to neutralize lysed cell suspension while vortexing. To measure malonyl-CoA concentration, we collected and chilled the supernatant after centrifuging. A 0.45 µm cellulose membrane (Sangon Biotech, Shanghai, China) was used to filter 3 mL of the same culture. Distilled water was used to wash the resulting cellulose membrane, followed by drying in ovens to determine dry cell weight, which was calculated by measuring the weight difference between empty membranes and those with cells. Malonyl-CoA concentration was measured based on our previous report^17^ by Liquid Chromatography-Mass Spectrophotometer (Shimadzu, Kyoto, Japan). A reverse-phase Gemini NX-C18 column (5 × 110 mm) and an electrospray ionization (ESI) source were employed.

## Electronic supplementary material

Supplementary information
