# Supplementary material for: Rational modular design of metabolic network for efficient production of plant polyphenol pinosylvin
Source: Sci Rep. 2017 May 3;7:1459. doi: 10.1038/s41598-017-01700-9 (PMC5431097; doi:10.1038/s41598-017-01700-9)
Supplement: Supplementary file 1 — Supplementary information [file 41598_2017_1700_MOESM1_ESM.doc]

**Rational modular design of metabolic network for efficient production of plant polyphenol pinosylvin**

Junjun Wu^1,2^, Xia Zhang^1,2^, Yingjie Zhu^1,2^, Qinyu Tan^1,2^, Jiacheng He^1,2^, Mingsheng Dong^1,2*^

^1^ College of Food Science and Technology, Nanjing Agricultural University, Nanjing, Jiangsu 210095, China.

^2^ Institute of Agro-Product Processing, Jiangsu Academy of Agricultural Sciences, Nanjing, Jiangsu 210095, China.

^*^ Corresponding author:

Mingsheng Dong

Mailing address: College of Food Science and Technology, Nanjing Agricultural University, 1 Weigang Road, Nanjing, Jiangsu, P.R. China

Phone: +86 25 84396989, Fax: +86 25 84399090

E-mail: [dongms@njau.edu.cn](mailto:dongms@njau.edu.cn)

**DNA sequences of optimized genes.**

***Rhodotorula glutinis* tyrosine ammonia lyase (TAL)**

atggcgccgcgcccgacttctcaaagccaggcccgcacttgcccgaccacccaggttacccaagttgatatcgttgagaaaatgctggcggctccgactgatagcaccctggagctggacggttatagcctgaacctgggtgatgttgtgagcgctgcgcgtaagggtcgtccggttcgtgttaaagatagcgatgaaatccgcagcaaaatcgacaagagcgttgaatttctgcgcagccaactgagcatgtctgtttacggtgtgaccaccggctttggcggctccgcggacacccgcaccgaggacgcaattagcctgcaaaaggcgctgctggaacaccagctgtgtggtgtgctgccgagcagcttcgacagctttcgcctgggtcgtggtctggagaacagcctgccgctggaagttgttcgcggtgcaatgaccattcgtgtgaactctctgacccgtggccatagcgctgttcgtctggttgttctggaagcactgaccaactttctgaaccacggtattaccccgattgttccgctgcgcggtaccatctccgcgagcggcgatctgtctccactgtcttacattgcagcggcgattagcggtcacccggatagcaaagttcacgtggttcatgaaggcaaagagaagatcctgtacgcgcgcgaagcgatggcgctgtttaacctggagccggtggttctgggtccgaaggagggcctgggtctggtgaacggtaccgcagtttccgcgagcatggcaaccctggcactgcacgacgcgcacatgctgagcctgctgagccaatctctgaccgcgatgaccgtggaggcgatggttggtcacgcgggcagcttccatccattcctgcacgatgttacccgtccgcacccgacccaaatcgaggttgcgggtaacattcgcaaactgctggagggctctcgcttcgcggttcaccacgaggaagaggttaaggttaaggatgatgaaggcattctgcgtcaggatcgttatccgctgcgcaccagcccgcaatggctgggtccgctggtgtccgacctgattcacgctcatgccgttctgaccatcgaagcgggtcaaagcaccaccgataacccactgatcgatgttgagaacaagaccagccatcacggtggcaactttcaagcggcagcggttgccaacactatggaaaagacccgtctgggcctggcccaaatcggtaaactgaacttcacccagctgaccgagatgctgaacgcgggcatgaaccgtggcctgccgagctgcctggcggctgaagacccatccctgagctatcattgcaaaggtctggacattgcggcggctgcatataccagcgaactgggccacctggctaacccggttaccacccacgttcaaccggctgaaatggcaaaccaggcggtgaacagcctggcgctgattagcgcacgtcgtaccaccgaatctaacgacgttctgtccctgctgctggcaacccacctgtactgcgtgctgcaggcgatcgacctgcgtgcgattgagttcgagttcaagaaacagtttggtccggccattgttagcctgatcgaccaacactttggtagcgcgatgaccggtagcaacctgcgtgatgagctggttgaaaaggttaacaagactctggccaagcgtctggagcaaaccaacagctacgatctggttccgcgctggcacgacgcttttagcttcgctgcaggcactgttgttgaggttctgtccagcaccagcctgagcctggcggccgtgaacgcatggaaggttgcggcagccgagagcgcgatctccctgacccgccaggttcgtgaaaccttttggtccgctgcaagcacctccagcccggcgctgtcttacctgagcccgcgcacccagatcctgtacgcatttgtgcgtgaggaactgggtgttaaagcccgccgtggtgacgttttcctgggtaaacaagaagttaccatcggcagcaacgttagcaagatttacgaagccatcaagagcggccgtatcaacaacgttctgctgaagatgctggcataa

***Petroselinum crispum* 4-coumarate:CoA ligase (4CL)**

atgggtgactgcgttgccccgaaagaggatctgatcttccgcagcaaactgccggacatttacattccaaagcatctgccgctgcatacctattgttttgagaacatcagcaaggttggcgacaagagctgtctgatcaacggcgcaaccggcgaaacctttacctacagccaggttgagctgctgtcccgtaaagttgccagcggcctgaacaagctgggcattcaacaaggtgataccattatgctgctgctgccgaactccccggagtactttttcgctttcctgggtgcgagctatcgcggtgcaatcagcactatggcgaacccattctttaccagcgcagaagtgatcaagcaactgaaagcgagccaagcgaagctgattatcacccaggcatgctatgttgacaaggttaaggactacgcagcggagaaaaacatccagatcatttgtattgacgatgcaccgcaggattgcctgcactttagcaagctgatggaagcggatgagagcgaaatgccggaagtggttattaacagcgatgatgtggtggcactgccgtacagctctggcaccaccggcctgccgaaaggcgttatgctgacccacaagggtctggttaccagcgttgcacaacaggtggatggtgataacccgaacctgtatatgcactccgaggatgttatgatctgcatcctgccactgttccatatctatagcctgaacgctgttctgtgttgtggtctgcgtgcgggcgttaccattctgatcatgcaaaagttcgacattgtgccgtttctggagctgattcagaagtataaggttaccattggtccgtttgttccgccgatcgtgctggccatcgcgaaaagcccggttgttgacaagtacgacctgtctagcgtgcgcaccgttatgagcggtgcagcgccgctgggtaaagagctggaggacgctgttcgtgcgaaattcccgaacgcgaagctgggtcaaggctatggcatgaccgaagccggtccggttctggcgatgtgtctggcgttcgccaaagagccgtatgagattaagtctggcgcatgcggtaccgttgtgcgtaacgccgagatgaaaatcgttgacccagaaaccaacgcgtctctgccgcgtaaccagcgtggtgagatttgcatccgtggtgatcagattatgaaaggttacctgaacgacccggaaagcacccgcaccaccatcgacgaagagggttggctgcacaccggtgacattggtttcatcgacgatgacgatgaactgttcattgttgatcgtctgaaagaaatcattaagtacaaaggttttcaagttgctccggcggagctggaagcactgctgctgacccacccgaccatcagcgatgccgcggtggttccgatgattgacgagaaagcgggtgaagtgccagtggcgtttgttgtgcgtaccaacggttttaccaccaccgaagaagaaatcaaacaatttgtgagcaaacaggttgtgttctacaaacgtatcttccgcgttttcttcgttgacgctattccgaaatccccgagcggcaagattctgcgtaaggatctgcgcgctcgtattgcgagcggcgacctgccgaagtaa

***Vitis vinifera* stilbene synthase (STS)**

atggcaagcgttgaagaaattcgtaatgctcagcgtgcaaaaggcccggcgaccatcctggcgattggcacggcgaccccggaccactgcgtctatcagagcgattacgccgactattactttcgtgtgaccaaaagtgaacacatgtccgaactgaaaaagaaattcaaccgtatttgtgataagtcaatgattaagaaacgctatatccatctgaccgaagaaatgctggaagaacacccgaacattggcgcttacatggcgccgtcgctgaatatccgccaggaaattatcacggctgaagtgccgaaactgggcaaggaagcggccctgaaagcgctgaaggaatggggtcaaccgaaaagcaagatcacccatctggtcttttgcacggcatctggtgtggaaatgccgggtgcagattataaactggcaaatctgctgggcctggaaaccagcgtgcgtcgcgttatgctgtatcaccagggttgctacgccggcggtaccgtgctgcgtacggctaaagatctggcggaaaacaatgcaggcgctcgcgttctggtggtttgtagcgaaattaccgtcgtgacgtttcgtggcccgagtgaagatgccctggactccctggtcggtcaagcactgttcggcgatggttctgcagctgttattgtcggtagcgatccggacgtgtctatcgaacgtccgctgtttcagctggtttcagcggcccaaaccttcattccgaactcggcaggtgcaatcgcaggtaatctgcgtgaagttggcctgacctttcatctgtggccgaacgttccgacgctgattagtgaaaatgtcgaaaaatgcctgacgcaggcatttgatccgctgggtatctcagactggaactcgctgttctggattgctcacccgggcggtccggcaatcctggatgcagtggaagcaaaactgaacctggacaagaaaaagctggaagccacccgtcatgttctgagtgaatacggcaatatgagctctgcatgtgtcctgttcattctggatgaaatgcgcaaaaagtcccacaaaggtgaaaaggcaaccacgggtgaaggtctggactggggcgttctgtttggtttcggtccgggtctgacgattgaaacggtggtcctgcatagcattccgatggttacgaactga

**Gene sequence of dCas9 protein**

atggacaaaaaatacagcatcggtctggcaatcggtacgaactctgttggctgggcggttatcactgatgaatacaaagtcccgtccaaaaaattcaaagtgctgggcaacaccgatcgtcactccatcaagaaaaacctgattggcgcgctgctgttcgactccggtgaaactgctgaagcgacccgcctgaaacgtactgcacgccgtcgttacacccgccgtaagaaccgcatttgctacctgcaggaaattttcagcaacgaaatggcgaaagtagacgacagctttttccaccgtctggaggagagctttctggttgaagaagacaaaaaacacgagcgccaccctattttcggcaacattgtggacgaagtggcataccacgaaaaatacccgaccatctaccacctgcgtaaaaaactggttgatagcaccgacaaagcagacctgcgtctgatctacctggcgctggcccacatgatcaaattccgtggccacttcctgatcgaaggcgatctgaacccagacaactctgacgtggacaaactgtttatccagctggtgcagacctataaccagctgtttgaagagaacccgatcaacgcttctggcgttgatgctaaagccatcctgtctgctcgtctgtctaaatcccgtcgtctggagaatctgattgctcagctgccgggcgaaaagaaaaacggtctgtttggcaacctgatcgcgctgtccctgggcctgactccgaacttcaaatctaacttcgacctggctgaagatgcaaaactgcaactgtccaaagacacttatgacgatgatctggataacctgctggcgcagatcggtgatcagtatgcagacctgttcctggctgccaaaaacctgtctgatgctatcctgctgagcgacatcctgcgcgttaacactgaaatcaccaaagctccgctgtctgcgtctatgatcaaacgctatgacgaacatcaccaggacctgaccctgctgaaagcgctggtacgtcagcaactgccggaaaaatacaaagaaatcttcttcgaccagagcaaaaacggctacgcgggttatatcgatggcggtgctagccaggaagagttctacaaattcatcaagccgatcctggaaaaaatggatggtactgaagagctgctggttaaactgaaccgtgaagacctgctgcgtaaacagcgtactttcgacaatggctctattccgcaccagattcatctgggtgaactgcatgcaattctgcgccgtcaggaggatttctatccgtttctgaaagataatcgcgaaaaaatcgaaaaaattctgactttccgtattccgtactacgtgggtccgctggcgcgtggtaattcccgctttgcatggatgacccgtaaatctgaagaaaccattaccccttggaacttcgaagaagtcgttgataaaggtgcttctgcacagtctttcatcgaacgcatgacgaatttcgacaaaaatctgccgaacgagaaagtactgccgaagcattccctgctgtacgaatacttcaccgtctataacgaactgacgaaagttaaatacgtaaccgaaggtatgcgcaaaccggcctttctgtccggtgagcagaagaaagccatcgtggacctgctgtttaaaaccaaccgcaaagtaaccgtaaaacaactgaaagaagactactttaagaaaatcgaatgttttgatagcgttgaaatttccggtgttgaagatcgtttcaacgcctctctgggcacgtatcacgatctgctgaagattatcaaagataaagactttctggacaacgaagagaacgaagacattctggaggacatcgttctgacgctgacgctgttcgaagatcgtgagatgatcgaagaacgtctgaaaacttacgctcatctgttcgacgataaagtcatgaaacagctgaaacgtcgtcgttatacgggttggggtcgcctgtctcgcaagctgattaacggcatccgcgataaacaatctggcaaaaccatcctggatttcctgaaaagcgatggcttcgcaaaccgtaacttcatgcagctgattcatgacgattctctgaccttcaaagaagatattcaaaaagcgcaagtttccggtcaaggcgactccctgcacgaacacatcgccaacctggcgggttctccagctatcaagaaaggcatcctgcaaactgtaaaagttgttgacgaactggttaaagttatgggccgtcacaaaccggaaaacatcgtgattgaaatggcacgtgagaaccagaccacccagaaaggtcagaaaaactctcgtgaacgtatgaaacgcattgaagaaggtatcaaagagctgggctcccaaatcctgaaagaacacccggtcgaaaacactcagctgcagaacgaaaaactgtacctgtattacctgcagaatggtcgtgacatgtatgttgaccaggaactggacatcaaccgtctgtccgactacgacgtcgacgcaatcgtgccgcagtctttcctgaaggatgactctatcgacaacaaagttctgactcgcagcgataaaaaccgcggcaaaagcgataacgttccgtccgaagaagttgtgaaaaagatgaaaaactattggcgtcagctgctgaatgccaaactgattacccagcgcaaatttgataacctgaccaaagcggaacgtggtggtctgtccgaactggataaagcaggtttcattaaacgtcagctggtagagacgcgtcagatcactaagcacgtggctcagatcctggactctcgtatgaacaccaaatatgacgaaaatgataagctgattcgtgaagtaaaggtgatcactctgaaaagcaagctggtctccgatttccgcaaagatttccagttttacaaagtgcgtgagatcaacaactaccaccatgcgcacgatgcgtatctgaacgctgtcgttggcaccgcactgatcaagaaatacccaaagctggaaagcgagttcgtgtatggtgattataaagtgtatgacgtacgtaaaatgatcgcgaagtctgaacaggaaatcggcaaagctaccgccaagtacttcttttactccaacattatgaacttcttcaaaaccgaaatcaccctggctaatggcgagatccgcaagcgccctctgattgaaactaacggtgaaaccggcgaaatcgtatgggataaaggtcgcgatttcgcgacggtacgtaaagtcctgtccatgccgcaggttaacattgttaaaaagaccgaagttcagaccggtggtttttccaaagaatccatcctgccgaaacgtaacagcgacaaactgatcgcccgcaaaaaggactgggatccaaagaaatacggtggtttcgactccccgaccgttgcttattctgttctggttgtggccaaagtggagaagggtaaaagcaagaaactgaaatctgttaaagaactgctgggcatcaccatcatggagcgtagctcctttgagaaaaaccctattgacttcctggaagcaaaaggctacaaagaagtaaagaaggacctgatcattaaactgccgaaatatagcctgttcgaactggaaaacggtcgtaaacgtatgctggcatctgcgggcgaactgcagaaaggcaacgaactggctctgccttctaaatacgtgaacttcctgtacctggcgtctcattacgaaaagctgaaaggcagcccagaggataacgagcaaaagcagctgttcgtggaacagcacaaacactacctggatgagatcattgaacagatctccgagttctctaaacgtgtaatcctggcggacgcgaatctggacaaagtactgtccgcatacaataaacaccgtgataaaccgatccgtgaacaggctgagaacatcatccatctgttcactctgactaacctgggcgcgccggctgcatttaagtacttcgacaccaccatcgatcgtaagcgttacactagcaccaaagaagtgctggacgcgaccctgattcaccagagcatcactggtctgtacgaaacccgcattgatctgtctcagctgggtggtgactaa

**Gene sequence of sgRNA chimera**

tgtacactgcaggtcgtaaatcactgcataattcgtgtcgctcaaggcgcactcccgttctggataatgttttttgcgccgacatcataacggttctggcaaatattctgaaatgagctgttgacaattaatcatccggctcgtataatgtgtggaattgtgagcggataacaatttcAGGAGACAACATGCCCAGTCGTTTTAGAGCTAGAAATAGCAAGTTAAAATAAGGCTAGTCCGTTATCAACTTGAAAAAGTGGCACCGAGTCGGTGCTTTTTT
